# Cost-Effective Mitigation of Greenhouse Gas Emissions in the Agriculture of Aragon, Spain

**DOI:** 10.3390/ijerph18031084

**Published:** 2021-01-26

**Authors:** Safa Baccour, Jose Albiac, Taher Kahil

**Affiliations:** 1 Department of Agricultural Economics, CITA-IA2, 50059 Saragossa, Spain; baccour.safa@gmail.com; 2 International Institute for Applied Systems Analysis (IIASA), A-2361 Laxenburg, Austria; kahil@iiasa.ac.at

**Keywords:** climate change, mitigation measures, biophysical processes, cost-efficiency, abatement costs, transaction costs, policy scenarios

## Abstract

Climate change represents a serious threat to life in earth. Agriculture releases significant emissions of greenhouse gases (GHG), but also offers low-cost opportunities to mitigate GHG emissions. This paper assesses agricultural GHG emissions in Aragon, one important and representative region for agriculture in Spain. The Marginal Abatement Cost Curve (MACC) approach is used to analyze the abatement potential and cost-efficiency of mitigation measures under several scenarios, with and without taking into account the interaction among measures and their transaction costs. The assessment identifies the environmental and economic outcomes of different combinations of measures, including crop, livestock and forest measures. Some of these measures are win-win, with pollution abatement at negative costs to farmers. Moreover, we develop future mitigation scenarios for agriculture toward the year 2050. Results highlight the trade-offs and synergies between the economic and environmental outcomes of mitigation measures. The biophysical processes underlying mitigation efforts are assessed taking into account the significant effects of interactions between measures. Interactions reduce the abatement potential and worsen the cost-efficiency of measures. The inclusion of transaction costs provides a better ranking of measures and a more accurate estimation of implementation costs. The scenario analysis shows how the combinations of measures could reduce emissions by up to 75% and promote sustainable agriculture in the future.

## 1. Introduction

Climate change is the consequence of massive greenhouse gas (GHG) emissions from human activities. These emissions are driven by many factors such as the growth of population and economic activities, the use of fossil fuels, the changes in land use (urbanization, deforestation, desertification), and the intensification of agriculture [1]. The high concentrations of GHG in the atmosphere absorb infrared radiation and are responsible for the increase of one degree in global annual temperatures between 1960 and 2017 [2,3]. GHG emissions are modifying the global climate system, with future predictions of higher temperatures, lower rainfall in arid and semi-arid regions, rise in the sea level, and higher frequency and intensity of extreme weather events [4,5]. Climate variability is affecting the availability and quality of water and water dependent ecosystems. Freshwater and marine species are modifying their geographic distribution areas and their seasonal activities, increasing the risk of extinction. Liu et al. [6] show also the importance of understanding changing water temperatures in rivers for addressing good riverine environmental management. The increase of anthropogenic GHG emissions affects the distribution of precipitation and modifies fluvial processes [7]. These climate change impacts are considered a serious threat to the sustainable development of human societies, and require immediate action [8]. Many scientists indicate that anthropogenic GHG pollution is one of the greatest threats of our time [9,10].

The United Nations Framework Convention on Climate Change (UNFCCC) was created in 1992 to respond to the threat of climate change, and the UNFCCC developed the Kyoto Protocol in 1997. However, the effect of this protocol on the reduction of global emissions has been only marginal. The latest policy initiative has been the Paris Agreement of 2015, which aims to ensure that global warming does not exceed 1.5 °C, so limiting the risks and impacts of climate change. This Agreement makes it clear that the global community must address the effects of climate change on agriculture in order to guarantee global food security [11]. In Europe, the concern for the environment has increased in recent years leading to ambitious abatement goals with GHG reductions of 20% in 2020, 40% being increased to 55% in 2030, and 80% in 2050.

Agriculture and global food security will be important issues in the coming decades, because of population increase and the expected impacts of climate change. The agricultural sector is important for food security in all countries, but also generates negative impacts on the environment and is responsible for 13.5% of global GHG emissions [3]. The goal is to mitigate climate change and ensure the reduction of GHG emissions into the atmosphere to avert their dangerous effects and promote the sustainable management of natural resources. The scope of this study is to identify cost-efficient mitigation measures in agriculture, while considering also the transaction costs of implementation and enforcement.

In Spain, the agricultural sector emits 10% of total emissions in the country and is an important source of non-CO_2_ emissions [12]. Agricultural and forestry activities are a source of low-cost opportunities to mitigate these emissions compared to other economic sectors. Soil carbon sequestration is a strategy that can be applied at widespread scale with a large potential to slow down global warming, mitigate GHG emissions, and reduce the concentration of CO_2_ in the atmosphere [13,14]. In addition, the enhancement of natural carbon sinks is considered as an important management tool to reduce atmospheric CO_2_ emissions [14]. Good forest management and better soil management can substantially reduce GHG emissions, by increasing carbon sequestration in soils and the amount of organic matter, and by adjusting the soil nitrogen cycle. The control of nitrogen entry into the soil is also a good practice to reduce direct and indirect N_2_O emissions and nitrate content in water bodies. Measures related to nitrogen fertilization management improve the efficiency of nitrogen use. These practices improve soil fertility, optimize crop productivity [15], and provide greater biodiversity and less erosion, runoff and pollution loads to the atmosphere and water media. Therefore, these practices are relevant to decision makers for the design of sustainable policies.

Sustainable agriculture, food security and the well-being of farmers require an integrated analysis of the performance of the agricultural sector. Several studies have investigated the problem of GHG emissions in agriculture at local, regional and global levels, assessing a wide range of mitigation measures [16,17,18,19,20,21]. This paper analyzes the underlying biophysical processes in order to evaluate different policy measures for mitigating GHG emissions and for reducing the social and environmental impacts of climate change. To reach this objective, we analyze a nonpoint pollution problem located in northeastern Spain (Aragon) looking at the abatement potential and the cost-efficiency of different mitigation measures. The abatement measures are evaluated individually and in combination using the Marginal Abatement Cost Curve (MACC) approach. The MACC approach is based on two pieces of information: the reduction of GHG emissions in CO_2_ equivalent, and the cost-efficiency in Euros per CO_2_ equivalent for each measure. Then, the different measures are ordered according to their cost-efficiency. The abatement achieved is the sum of the abatement of the sequence of best measures selected by their cost-efficiency. This procedure provides decision makers with the increasing costs incurred in reaching higher abatement targets.

We develop also several mitigation policy scenarios up to 2050 for agriculture to assess the impacts of these policies on the balance of GHG emissions. Climate change and agricultural nonpoint pollution are global problems that affect all regions in the world, but it is important to analyze the problem locally in order to gain knowledge of the best alternatives for atmospheric and water pollution abatement in each zone. The purpose is to promote awareness and mobilize society to confront climate change. The assessment of the environmental, political, economic and social impacts in each region is a precondition to confront climate change, global warming, and pollution problems with efficient measures adapted to local specific conditions. The MACC approach has been used to analyze the efficiency of mitigation measures in different situations with and without taking into account transaction costs and the interaction between measures, by estimating the environmental and economic outcomes.

The contribution of this study to the literature is to provide a detailed assessment of GHG mitigation measures in agriculture and forestry. The outcomes from these measures include the enhancement of soil carbon sequestration, efficiency gains in the use of nitrogen and water, improvements in livestock digestion, and reduced nitrogen pollution loads from crops and livestock. Furthermore, the consideration of transaction costs is included in the analysis providing a better ranking of measures and a more accurate estimation of implementation costs. In addition, the estimation of the MACC for individual measures has been extended to the combination of measures, where measures interact with each other.

This information can contribute to the ongoing policy discussion and the improvement of decision-making on mitigation. The results of this paper highlight the importance of including transaction cost for a more reliable appraisal of the costs of implementation. Our results indicate also that the interaction between measures reduce the abatement rate of subsequent measures and worsens their cost-efficiency, especially for measures with positive costs. The large differences between the efficiency of individual or combined measures show the importance of considering the interaction of measures for policy design.

The paper is organized as follows. Section 2 presents a general description of the study area and the main agricultural activities in the region. Section 3 describes the main biophysical processes in agricultural nonpoint pollution and the abatement alternatives. Then the MACC of measures is developed and the abatement scenarios up to 2050 are described. Section 4 presents the main results of MACC for individual and combined measures, and the impacts on the GHG balance of emissions in 2050. Section 5 summarizes the main conclusions.

## 2. Study Area

In this study, Aragon is an illustrative case, and the results from its mitigation measures and policy scenarios could provide a relevant insight for other regions in countries confronting high levels of agricultural nonpoint pollution into the atmosphere and water media. Aragon is an interesting case to evaluate climate change mitigation efforts in agriculture. It is a large region, with its land divided equally between cropland and forested areas, and with an important livestock sector. The GHG emission from agriculture represents a quarter of the total emissions in the region, double the percentage of agricultural emissions at national or international levels. The main agricultural nonpoint pollution loads come from the substantial acreage of irrigated crops (400,000 ha) and the extensive herds of swine and sheep (1.4 million Livestock Unit equivalent). An advantage in the region is the large area of forests, which provides opportunities for management techniques oriented to carbon sequestration. Agricultural pollution is also degrading the quality of water bodies, and 4% of the drinking water supply areas are not complying with water quality standards because of excessive nitrate levels, with an affected population of 12,000 inhabitants [22,23]. In addition, the potential impact of climate change differs across Europe, but it is especially strong in the south of Europe where countries are more vulnerable [24].

The State of Aragon is located in northeastern Spain in the Middle Ebro Valley, covering an area close to 48,000 km^2^ [25]. Most land is for agricultural and forestry use, with 49% being agricultural and 50% woodland and forested areas covered with vegetation, open and closed forest. The use of land in agriculture is characterized by an extensive rainfed area (66%), coupled with a substantial irrigated area (34%). The major cultivated crop acreages are barley (55%) and wheat (27%) in dryland, and barley (27%), corn (22%) and alfalfa (20%) on irrigated land. The main irrigation systems are surface irrigation (47%) followed by sprinkle in arable crops (38%) and drip in woody crops (15%) [26]. Vineyards, olive and almond trees are the most important fruit-trees in the region in terms of area and profitability. Livestock production represents 63% of the net income in the agricultural sector in Aragon and crop production 34%. The swine sector has a large economic and social importance representing 62% of livestock production and 36% of agricultural production [27].

The forested area is located in Huesca (37%), Teruel (37%) and Zaragoza (26%). Carbon sequestration by forests is an important factor to combat climate change since forests capture close to 3 MtCO_2_e, which could be valued at 116 M€ using the estimate of 40 €/tCO_2_e for the social cost of carbon of the OECD [28]. The species that fix the largest amount of carbon are pine forests (38%), followed by oak (19%).

**Figure 1:**
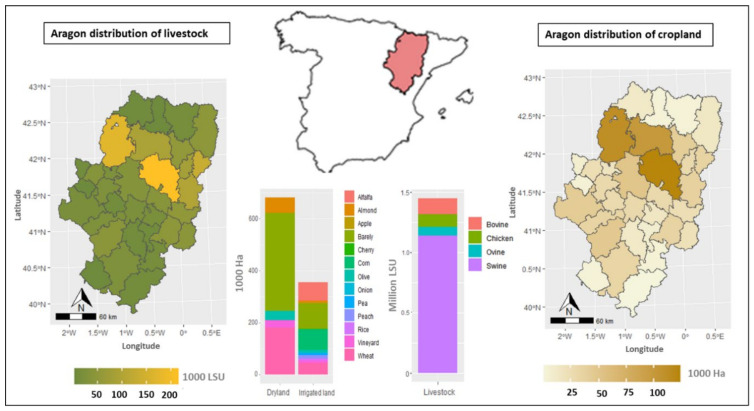
Main field crops, fruit trees and livestock herds. (LSU = Livestock Unit equivalent).

The types of crop and animal species analyzed in this study have been selected by their level of activity and profitability. The selected crops are wheat, barley, almonds, olives, and vineyard cultivated in dryland and irrigated land, and alfalfa, maize, onion, rice, pea, apple, peach, and cherry cultivated only in irrigated lands. These crops represent 65% of the total crop area in the region and generate 490 M€ in farmers’ benefits. The selected livestock species are cattle, sheep, pigs and chicken with 2100 M€ in benefits to farmers (Figure 1).

## 3. Materials and Methods

The methodology to estimate agricultural GHG emissions is the Level 1 procedure of the IPCC method (Intergovernmental Panel on Climate Change, Tier 1). The main source of non-CO_2_ emissions considered in this study are direct and indirect nitrous oxides emissions (N_2_O) from fertilizers, methane (CH_4_) from enteric fermentation, and N_2_O and CH_4_ from manure management. The procedure consists in multiplying the emission factors (EF) by the specific data of the region, which are surface of crop *i* ($X_{i}$), heads of livestock $j\;\left(C_{j}\right)$, fertilization ($N_{i}$), nitrogen leaching ($L_{i}$), and nitrogen excreted by each animal *j* ($Nex_{j}$) [29,30]. The databases used in this study are from 2014 because of the availability of data disaggregated at municipal level, covering cropland (dry and irrigated), livestock census, and irrigation systems. The emission factors for N_2_O emissions from agricultural soils are 0.0125 Kg of N_2_O-N per kilogram of nitrogen input for direct emissions ($EF_{1}$), and 0.025 Kg of N_2_O-N per kilogram of N leached for indirect emissions ($EF_{2}$). The emission factors for CH_4_ livestock emissions are taken from the European Environment Agency [31] and classified by animal type *j*. The emission factors for N_2_O emissions from manure management are taken from [32] and classified by the manure management systems *k*. The emissions are given by the following equations:

(1) $$\mathrm{Direct}\;\mathrm{emissions}\;\mathrm{of}\;N_{2}O=\sum_{i=1}^{n}(N_{i}\cdot X_{i}\cdot EF_{1}.(44/28)\cdot WP_{N_{2}O})/1000$$

(2) $$\mathrm{Indirect}\;\mathrm{emissions}\;\mathrm{of}\;N_{2}O=\sum_{i=1}^{n}(L_{i}\cdot X_{i}\cdot EF_{2}\cdot (44/28)\cdot WP_{N_{2}O})/1000$$

(3) $${\mathrm{CH}}_{4}\;\mathrm{from}\;\mathrm{enteric}\;\mathrm{fermentation}=\sum_{j=1}^{n}(C_{J}\cdot EF_{3j}\cdot WP_{CH_{4}})/1000$$

(4) $$N_{2}O\;\mathrm{emissions}\;\mathrm{from}\;\mathrm{manure}\;\mathrm{management}=\sum_{j,k}^{n}(C_{j}\cdot Nex_{j}\cdot EF_{4k}\cdot (44/28)\cdot WP_{N_{2}O})/1000$$

(5) $${\mathrm{CH}}_{4}\;\mathrm{emissions}\;\mathrm{from}\;\mathrm{manure}\;\mathrm{management}=\sum_{j=1}^{n}(C_{J}\cdot EF_{5j}\cdot WP_{CH_{4}})/1000$$

The coefficient (44/28) is the molecular weight ratio between N_2_O and nitrogen (N_2_), and WP is the warming potential of the greenhouse effect for CH_4_ and N_2_O.

The approach used to estimate the abatement potential from individual measures and from combined measures (taking into account interaction between measures) is the marginal abatement cost curve (MACC). This tool is highly appropriate for a reliable assessment of mitigation policies. The calculation of the balance of GHG emissions and the cost-efficiency of measures is performed evaluating the economic and environmental outcomes from agriculture in each municipal district.

The methodology used to estimate the potential for mitigation is the following: first, we select the most applicable and efficient mitigation measures in Aragon and collect the information on the costs and efficiency of each measure *m*. The selection of measures is based on technical information and biophysical processes such as carbon sequestration in soils, the efficiency of nitrogen and water use, and the reduction of nitrogen excreted from livestock. The description and selection of mitigation measures are presented below. In addition, we analyze the adoption and degree of applicability of selected measures in the region depending on land use data and experts’ judgement.

Second, the abatement potential of each measure *m* is determined from the abatement rate $AR_{mi}\;\mathrm{or}\;AR_{mj}$ of each crop *i* or each species of animal *j*, and then the abatement rates are multiplied by the acreage $X_{i}$ or animal heads $H_{j}$ covered by the measure (Equation (6)). $PC_{m}$ is the private cost of each measure *m* that represents the difference between the benefits $B_{m}$ (increase in yields or decrease in production costs) and the costs of implementing measure $C_{m}$. Equation (8) represents the cost-efficiency of selected mitigation measures.

(6) $$AP_{m}=\underset{m}{\sum }AR_{mi}\cdot X_{i}\;or\;\underset{m}{\sum }AR_{mj}\cdot H_{j}$$

(7) $$PC_{m}=B_{m}-C_{m}$$

(8) $$CE_{m}=PC_{m}/AP_{m}$$

Finally, the different measures are classified according to their cost-efficiency using the marginal abatement cost curve (MACC) method. A description of MACC and the main characteristics and details of implementation of the curve are presented in Section 3.2. The MACC calculation is further developed by including the transaction costs. We follow the same steps described above, but now the costs of measures include both the implementation costs $C_{m}$ and the transaction costs $TC_{m}$. Equation (9) defines the measure costs $MC_{m}$ given by the difference between the benefits $B_{m}$ and costs of the measure *m* $\left(C_{m}+TC_{m}\right)$.

(9) $$MC_{m}=B_{m}-(C_{m}+TC_{m})$$

The transaction cost is the cost of implementation and enforcement mechanisms that ensure the adoption of mitigation measures by farmers, and other design costs incurred because of the strategic behavior of farmers or other stakeholders and interest groups. The transaction costs are further discussed in Section 3.3.

The estimation of the abatement potential of combined measures by taking into account the interaction between measures is challenging given the lack of technical and economic information when measures are taken simultaneously. Measures’ interaction should be included in the MACC analysis because of the changes in abatement potential and cost-efficiency from interaction. For example, optimizing nitrogen fertilization reduces nitrogen fertilizer use and leaching, and therefore less abatement will be achieved by other measures, reducing the efficacy of subsequent measures. Moran et al. and Schulte et al. [33,34] evaluate the interaction between measures by adjusting their abatement potential when calculating the cumulative abatement. In this study, the abatement potential of combined measures is evaluated by ordering the sequence of measures, and then estimating the abatement potential and cost-efficiency of each measure by considering the order in the sequence and the interaction with previous measures. The order of measures is determined from the abatement potential of individual measures and the degree of applicability. The degree of applicability of measures come from the technical information provided by experts. The procedure generates two sequences of measures, one for crops and the other for livestock. The sequence of measures for crops is the following: M1. N input optimization; M2. Manure fertilization; M3. Minimum tillage; M4. Cover crops (arable crops); M5. Crop rotation; M6. Cover crops (woody crops); M7. Nitrification inhibitor; and M8. Irrigation modernization. The sequence of measures for livestock is the following: M1. Manure fertilization; M2. Protein diets; M3. Manure treatment plants; and M4. Fat additives.

In the mitigation scenarios for 2050, three scenarios are developed that differ in the degree of application of mitigation measures, taking into account the future evolution of agriculture and the uncertainty about the use of land and natural resources. The projections for the agricultural sector include reductions in the irrigated area of barley and wheat, which switch to dryland. This is based on a 12% decline in water availability estimated by the Centro de Estudios y Experimentación de Obras Públicas (CEDEX) [22] for the Ebro basin in the period 2040–2070. The water reduction especially affects barley and wheat, which are the less profitable irrigated crops. It is assumed that 25% of the barley and wheat irrigated acreage (36,000 ha) switches to dryland [35,36]. Besides, an increase of 30% in swine numbers is assumed given the previous large herd expansion in the region from 3.5 to 8 million heads between 2000 and 2019, but taking into account the overcrowding effects limiting further expansion. Three mitigation scenarios are considered. The first is business as usual with no mitigation measures engaged in this scenario (S1). The second scenario includes only the most cost-efficient mitigation measures (S2), which are optimizing nitrogen fertilization, manure substituting synthetic fertilization, and minimum tillage. These three measures are win-win measures since they abate emissions at negative costs (benefits) to farmers. The third scenario includes all mitigation measures (S3), described below. The procedure followed is to calculate the GHG emissions under each scenario, the abatement achieved and the ensuing abatement costs.

### 3.1. Evaluation and Selection of Mitigation Measures in Agriculture and Forestry

There is an existing set of measures considered in the literature to mitigate GHG emissions in agriculture. Asgedom and Kebreab [37] assess various mitigation measures for crop and livestock production systems under different biophysical scenarios. They show that these measures have numerous economic and environmental benefits in reducing and avoid GHG emissions. Soil management is important to abate N_2_O emissions and improve carbon sequestration by soils. Therefore, better soil management can substantially reduce GHG emissions, and increase carbon sequestration in soils through the absorption of CO_2_ by plants, increase organic matter in soils, and adjust the nitrogen cycle. These improvements promote greater fertility and productivity and enhanced soil biodiversity, while reducing erosion, runoff and pollution to the atmosphere and water media. Soil management practices include crop rotation [38], substitution of synthetic fertilizers by manure [39], use of efficient varieties with a larger mass of roots [40], cover crops [41], and reduced tillage or no-tillage [42]. Fertilization management is also a good alternative to reduce direct and indirect N_2_O emissions and the nitrogen loads from crops into water bodies. These measures can improve atmosphere and water quality and increase nitrogen and water use efficiency.

The application of technical interventions and structural changes in livestock production could reduce emissions, increase carbon sequestration in pastures, and support sustainable livestock production. Livestock management measures include improving pasture, intensifying ruminants’ diets, and changing breeds [43]. In addition, the use of CH_4_ inhibitors and fat in the diet of ruminants reduces CH_4_ emissions from enteric fermentation [44]. Manure management measures include manure storage and manure treatment plants based on biological processes [45]. Forest management for carbon storage in forests and shrubs is another option to mitigate the effects of climate change, while enhancing the provision of ecosystem services. Forest management measures increase biomass and carbon in forest stands by modifying the thinning regime, the rotation period, and the harvesting operations. These strategies could improve soil protection, reduce the risk of fire, promote biological stability, and increase the value of products [46,47].

This paper includes crop, livestock and forest measures. Crop measures include nitrogen input optimization, crop rotation with legumes, cover crops, efficient irrigation technologies, nitrification inhibitors, and minimum tillage. The measures for livestock are substitution of synthetic fertilizers by manure, manure treatment plants, use of fat additives in the diet of ruminants, and reduction of protein content in the diet of swine. In forestry, the focus is on management measures intended for carbon sequestration. The description of the selected measures is presented in Table 1.

### 3.2. The MACC Approach

The marginal abatement cost curve (MACC) was first used by [48] to analyze mitigation policies in US agriculture. Subsequently, it has been used in different studies at global, regional and national levels [20,21,49,50,51]. The MACC is a tool for mitigation policy analysis that brings a wide range of information about mitigation measures, and shows the potential of GHG abatement and the associated costs for different alternatives. This information reveals which are the most effective policy interventions in order to facilitate the exchange between scientific studies and policy decision-making.

The MACC is a figure with a series of discrete bars that represents the rising costs and the abatement of emissions from each mitigation measure. The width of each bar represents the reduction of GHG emissions (MtCO_2_e), while the height of the bar shows the cost-efficiency of the measure (€/tCO_2_e). The different measures are ordered according to their cost-efficiency, so that from left to right of the curve the cost-efficiency worsens as the accumulated abatement of measures increases and additional measures become more expensive. The figure has two parts, with the first part representing win-win measures that reduce emission and have negative costs, thus generating both economic and environmental benefits [52]. The second part of the figure represents measures with positive costs for stakeholders, and usually the cost burden falls on farmers. Therefore, the implementation of measures involves private costs for farmers, but generates environmental benefits for the whole of society. Within the two parts, we can find measures reducing GHG emissions and saving money, and others with higher reductions but requiring costly investments. The MACC approach identifies the most efficient mitigation measures and can be compared to reference threshold costs per tCO_2_e, such as the social cost of carbon from the OECD [28]. The MACC analysis is limited in different aspects. First, the choice of the emission categories considered in the analysis and the implementation costs included are determined by the researchers. Second, the MACC representation and cost-efficiencies are estimated at one point in time, but these estimates would be less accurate in the long run. MACC estimates become more uncertain in the future because of the charges in the implementation costs of current measures and from new measures embodying more advanced technologies. However, this analysis provides information to decision makers on the efficiency of measures that can be implemented in the short run. In this study, the MACC is used to analyze mitigation measures in individual and combined forms. The effect of individual measures is analyzed without taking into account the interactions and dependencies among them, while combined measures are analyzed considering potential interactions between them. We also evaluate mitigation measures by including their transaction costs.

### 3.3. Transaction Costs and Costs of Measures

In economics, transaction costs are defined as the costs incurred to complete economic exchanges or market transactions. Transaction costs were introduced to the literature by Coase in “The Nature of the Firm”, indicating the cost of using the price mechanism. Other researchers have developed the theory of transaction costs: Williamson states that they are the costs of operating the economic system [58], and Cheung [59] indicates that transaction costs are all the costs from exchanges, including the physical processes of production and transportation. Dahlman [60] states that transaction costs include research and information costs, negotiation and decision costs, and surveillance and execution costs. Transaction costs are also included in discussions on property rights and ecological and environmental policies. McCann [61] indicates that transaction costs should be considered in environmental policy design, and included in environmental and natural resources analysis. Garrick et al. [62] consider that transaction costs are useful to compare policy measures and these costs have to be included in the evaluation of the costs and benefits of policies. The estimation of transaction costs is challenging because of the different definitions and types of transaction costs. Liu and Shen [58] also indicate the difficulties in estimating the cost of non-market aspects such as transaction behaviors that differ according to culture and customs.

Many studies have measured the transaction costs of environmental policies. Howitt [63] shows that transaction costs represent 8% of water purchase costs in California. McCann and Easter [64] report that transaction costs amount to 38% of the total costs of the United States Program of Technical Assistance for Agriculture. Mettepenningen et al. [65] analyze the transaction costs of European agri-environmental schemes and indicate that transaction costs are about 15% of the total cost of the policy and about 25% of compensation payments. Coggan et al. [66] indicate that transaction costs of an environmental policy including both public and private transaction costs range from 20% to 50% of the total policy costs. Rorsted et al. [67] assess transaction costs of agricultural policies and demonstrate that transaction costs of environmental measures are about 20% of total policy costs. In this paper, based on the previous literature, we consider that transaction costs are 20% of the total cost of the measure. This is only an approximation because of the lack of information on the implementation and enforcement mechanisms in each mitigation measure to ensure adoption by farmers, and lack of information on the strategic behavior of farmers.

In this study, the costs of implementing measures and practices include the investment costs (seeds, machinery or equipment) and the costs of farm operations associated with each practice. In some cases, the costs are negative because the measures result in private benefits to farmers. Some examples are the costs saved from reductions in excessive fertilization, the increases in yields, or the substitution of synthetic by organic fertilization. The main sources of data used in the calculations of the costs are the regional statistical data published by the Spanish Ministry of Agriculture, literature reviews, and the outcomes from regional agro-economic models [68].

## 4. Results

### 4.1. Assessment of Agricultural GHG Emissions in Aragon

Agricultural activities generate important pollution loads from excessive nitrogen fertilization and intensive livestock (Table 2). The consequence is the degradation of ecosystems by emissions of nitrates to water media and ammonia to the atmosphere, and global warming from emissions of N_2_O and CH_4_ to the atmosphere.

**Figure 2:**
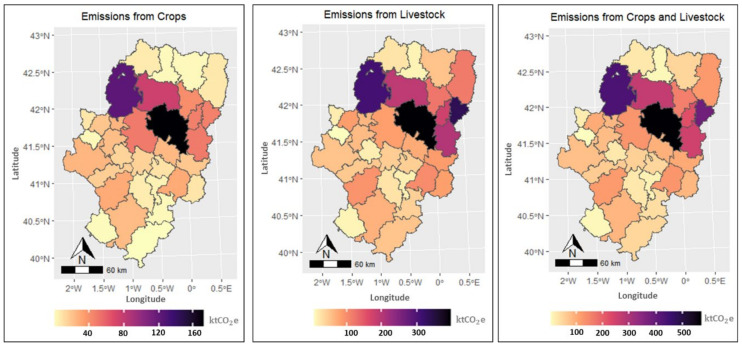
GHG emissions by county from crops, livestock, and the sum of crops and livestock.

**Figure 3:**
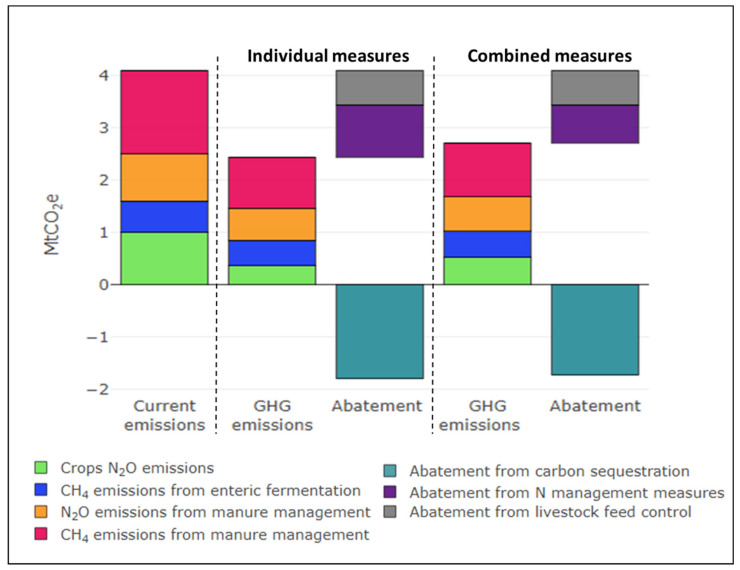
Agricultural GHG emissions and abatement under individual and combined measures. Individual measures are measures evaluated separately without considering interactions, and combined measures are evaluated taking into account their interactions.

In Aragon, agricultural GHG emissions amount to 4.1 MtCO_2_e, which represent 25% of the total emissions of the region. The emission loads are located mainly in the districts of Monegros (14%), Cinco Villas (11%), La Litera (9%) and Hoya de Huesca (6%), due to the concentration of irrigated crops and swine production in these areas (Figure 2). The emissions come mainly from CH_4_ and N_2_O loads from manure management, which amount to 1.6 MtCO_2_e and 0.9 MtCO_2_e, respectively. Direct and indirect emissions of N_2_O from nitrogen fertilization of crops are close to 1 MtCO_2_e, with 70% from direct emissions and 30% from indirect emissions of leaching and runoff. The largest share of fertilizer emissions come from irrigated crops (68%) and the rest from rainfed crops (32%), (Figure S1 in Supplementary Materials). Surface irrigation generates higher N_2_O emissions than sprinkler and drip irrigation systems, with surface irrigation accounting for 56% of emissions, sprinkler 40%, and drip 4%. The high N_2_O emissions from surface irrigation are explained by the higher N application compared to other irrigation systems and the efficiency of sprinkler and drip irrigation systems in reducing nitrogen leaching from crop activities. Emissions from enteric fermentation are 0.6 MtCO_2_e or 17% of agricultural emissions (Figure 3).

Our results indicate that the GHG abatement potential from all individual measures (with and without transaction costs) is 3.45 MtCO_2_e, which reduces current agricultural emissions by 84%. However, if the interaction among measures is taken into account, the reduction in emissions is only 75%. The large abatement of GHG emissions provided by measures in both cases indicate the potential synergies between improving agricultural productivity and mitigating GHG emissions. Carbon sequestration measures such as forest management, cover crop, minimum tillage and crop rotation have the highest GHG abatement potential with reductions of around 1.79 MtCO_2_e under both individual and combined measures. Measures that address livestock feed such as protein diet for swine and fat additives for ruminants have an abatement potential of about 0.65 MtCO_2_e. N management measures such as N optimization, manure fertilization, and nitrification inhibitors could reduce emissions by 1 MtCO_2_e with individual measures and 0.73 MtCO_2_e with combined measures (Figure 3). The results suggest that some practices such as N optimization, manure management, crop rotation, and protein diet have important win-win mitigation potential, because abatement is attained without additional costs for individual or combined measures. These measures increase both farmer’s net income and the GHG abatement level. Cui et al. [69] prove that enhanced agricultural management practices reduce the use and pollution loads of nitrogen fertilizers while increasing farmers’ income. Clark et al. [70] indicate that reducing GHG emissions from the food system can deliver additional benefits such as reducing nutrient and water pollution, decreasing land use change, and improving biodiversity outcomes.

### 4.2. Mitigation Measures Assessment and Transaction Costs

**Figure 4:**
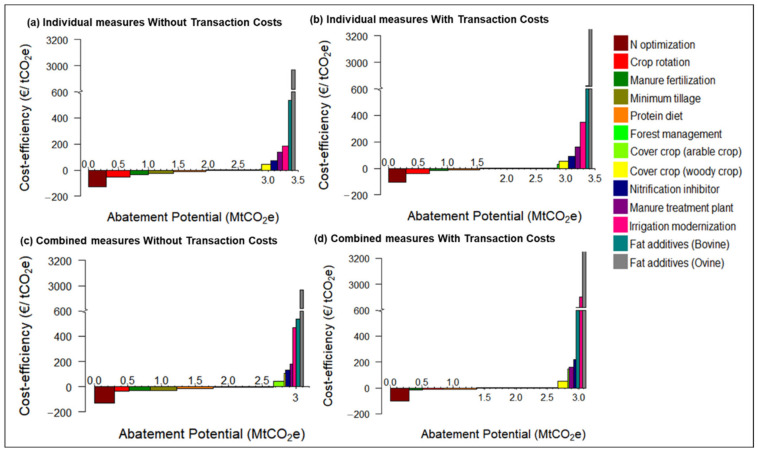
Marginal Abatement Cost Curve (MACC) of individual and combined measures.

Figure 4a,b shows the abatement potential and the cost-efficiency of individual measures with and without transaction costs. Results show that transaction costs worsen the cost-efficiency of measures without changing their abatement potential. Measures with negative abatement costs have significant abatement potential up to 2.9 MtCO_2_e for individual measures, although, including transaction costs, the abatement of measures that still have negative costs shrinks to 1.55 MtCO_2_e. Win-win measures for mitigating emissions are N optimization, crop rotation, manure fertilization, and improvement of swine feed. Transaction costs worsen cost-efficiency, and some win-win measures such as minimum tillage and forest management become less attractive when including transaction costs.

Measures improving nitrogen efficiency and reducing N loads to the atmosphere and water media are evaluated. The N optimization measure provides a significant abatement of about 0.3 MtCO_2_e at negative costs in line with the findings by [19,20,21,51]. This measure generates benefits to farmers that decrease their private costs while abating pollution and delivering environmental benefits. Manure fertilization substituting synthetic fertilization is another interesting measure achieving a 9% abatement at negative costs of −35 €/tCO_2_e and −17 €/tCO_2_e for the individual measure without and with transaction costs, respectively. Under this measure, the synthetic fertilization is reduced to 76,000 tN, decreasing pollution into water media (−3000 tNO_3_-N) and the direct and indirect N_2_O emissions (−218,000 and 97,000 tCO_2_e, respectively). This measure has substantial interest in our study area, because of the availability of manure that could cover the nitrogen needs of most crops, especially in large irrigation districts [71]. Pellerin et al. [21] point out that manure fertilization in France reduces N_2_O emissions, with a negative cost of −74 €/tCO_2_e. Albiac et al. [20] consider an increase in the proportion of organic fertilization of up to 40% and 55% in Spain, which implies a cost-efficiency level of 75 and 140 €/tCO_2_e, respectively. The application of this measure requires organizing cooperation between farmers cultivating crops and livestock producers.

Another measure considered is irrigation modernization, although advanced irrigation technologies require large investment and higher operating costs. Irrigation modernization improves efficiency in the use of water and fertilizers, which reduces water use by 15% and fertilizer use by 13%. Albiac et al. [20] also indicate that irrigation modernization in Spain could reduce emissions by 2.1 MtCO_2_e with a cost-efficiency of 400 €/tCO_2_e. Nitrification inhibitors are also a type of measure that requires expenses. This measure is quite efficient for maize, achieving an 84% reduction in N_2_O but costs are significant. Pellerin et al. [21] point out that nitrification inhibitors have a cost-efficiency of 60 €/tCO_2_e for individual measures, which is very close to our estimate.

Measures that increase carbon sequestration in crops and forests are also analyzed, such as crop rotation, minimum tillage, cover crops, and forest management. Conniff et al. [73] indicates that these measures increase carbon sinks with costs ranging from −35 to 88 €/tCO_2_e which are close to our results. Crop rotation with legumes is a measure with double benefits since it increases carbon sequestration and reduces N fertilization and N leaching. The abatement potential of crop rotation is 0.4 MtCO_2_e at negative cost of −53 €/tCO_2_e, although when transaction costs are considered the cost-efficiency worsens to −42 €/tCO_2_e. Minimum tillage is another interesting measure directed to increase carbon sequestration. This measure provides a significant abatement of close to 0.4 MtCO_2_e with negative costs of −27 €/tCO_2_e, although transaction costs worsen the cost-efficiency of the measure to 2 €/tCO_2_e. Macleod et al., Moran et al., and Sanchez et al. [19,49,50] indicate that minimum tillage has negative cost-efficiency. On the contrary, Pellerin et al. [21] indicate that minimum tillage in France has positive costs, although not significant (8 €/tCO_2_e).

Forest management to enhance carbon sequestration has an important role in mitigating climate change. This type of management increases carbon capture, with an abatement potential of 0.9 MtCO_2_e and costs at 0.5 €/tCO_2_e and −3.5 €/tCO_2_e when transaction costs are included or not, respectively. The negative cost is a consequence of the gains in income from enlarged wood sales.

Others measures assessed for reducing GHG emissions are manure treatment plants and improvements in the diet of animals, but results show that the costs of these measures are very high and require substantial investments. The implementation of these measures is difficult because they reduce the net income of farmers, and farmers will not implement them in the absence of public incentives.

The abatement potential of crop and livestock mitigation measures and forest carbon sequestration for each county of Aragon provide more detail about the efficiency of each mitigation measure in each location. Generally, the measures can be effective and viable in districts where agricultural activities are intense with large nonpoint pollution emissions (Figure S2).

### 4.3. Effect of Interaction between Measures

Results show that the abatement potential when all measures are combined is 3.11 MtCO_2_e, which reduces current emissions by 75%. Combined measures with negative abatement costs have significant abatement potential, and reduce emissions to 2.6 MtCO_2_e. The interaction between measures decreases the cumulative abatement potential by 10% compared to the abatement achieved with individual measures (When a measure is taken after other measures, the previous measures have reduced already the pollution stock and less pollution remains to be abated. The additional abatement of a subsequent measure is smaller than in the case of measures being taken individually [49]). In most cases, the interaction between measures reduces the abatement rate of subsequent measures and worsens the cost-efficiency, especially for measures with positive costs. For example, the abatement potential of irrigation modernization falls from 0.10 to 0.05 MtCO_2_e and the cost-efficiency worsens from 184 €/tCO_2_e to 470 €/tCO_2_e without including transaction costs, and from 346 €/tCO_2_e to 802 €/tCO_2_e including transaction costs. The abatement potential of nitrification inhibitors also decreases from 0.11 to 0.04 and the cost-efficiency worsens from 74 €/tCO_2_e to 182 €/tCO_2_e without transaction costs, and from 90 €/tCO_2_e to 220 €/tCO_2_e including transaction costs. (Figure 4c,d). Some measures such as forest management or fat additives for ruminants do not have interactions with previous measures and thus maintain their abatement potential and cost-efficiency. Macleod et al. [49] indicate that the large discrepancies between individual and combined measures indicate that interactions are important in developing MACC.

### 4.4. Mitigation Policy Scenarios

**Figure 5:**
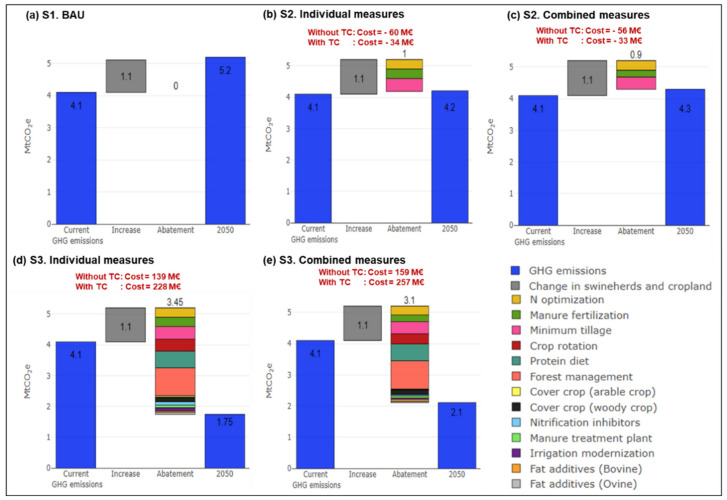
Mitigation policy scenarios for the 2050 horizon. The figure shows the current and the 2050 emissions under the different scenarios. “Increase” refers to the increase of emissions in 2050 from changes in swineherds and cropland, and “Abatement” refers to the reduction of emissions from measures. BAU is the Business as Usual scenario, and TC are transaction costs.

Climate change entails large uncertainties for the future development and sustainability of the agricultural sector. In a globalized world, mitigation and adaptation measures require coordination of countries and sectors, while ensuring at the same time food security for the human population. Mitigation scenarios show the consequence of policy decisions on agricultural GHG emissions in the future, linking the balance of GHG emissions to each policy choice. Future agricultural developments up to 2050 include the increase of swineherds and the reduction of water available for irrigation, with some barley and wheat production changing from irrigated to dryland. These future developments of agriculture in Aragon would increase emissions by 26% in the next 30 years compared to the current situation, with emissions reaching 5.2 MtCO_2_e in the business as usual scenario (S1). This increase is driven by the rise of GHG emissions from manure management (Figure 5a).

Under the scenario of implementation of the more viable and efficient measures (S2), the individual measures reduce GHG emissions only by 1 MtCO_2_e down to 4.2 MtCO_2_e in 2050 with negative costs (benefits) of 60 M€ to farmers. The consideration of transaction costs reduces these negative costs of farmers to 34 M€. Taking into account interactions, the combination of measures reduces emissions only by 0.9 MtCO_2_e with negative costs at 33 M€ or 56 M€ by including and not including transaction costs, respectively (Table S1). These negative costs or positive benefits will facilitate the smooth implementation of measures and the support from farmers. This scenario allows the dampening down of current GHG emissions up to 2050 under both individual and combined measures. However, the stabilization of GHG emissions is not a very ambitious alternative to confront the threat of climate change, conserve natural resources, and protect the environment (Figure 5b,c).

The implementation of all measures (S3) provides a significant abatement of 3.4 MtCO_2_e in agricultural emissions in the 2050 horizon, at a positive cost of 139 M€ without transaction costs and 228 M€ including transaction costs. When interactions are accounted for, abatement diminishes to 3.1 MtCO_2_e with a positive cost of 159 M€ without transaction costs and 257 M€ including transaction costs (Figure 5d,e; Table S2). In order to implement mitigation measures, the cooperation between agents and interest groups is needed for collective action. Albiac et al. [74] point out that the sustainable management of natural resources and the protection of the environment require a sufficient degree of collective action and cooperation among interest groups. Jiao et al. [75] emphasize the urgency of sharing knowledge and efforts among scientists, farmers, and institutions. Cooperation is an essential ingredient for the sustainable management of natural resources and the agricultural sector, the protection of ecosystems, and the well-being of future generations.

## 5. Discussion

The results of the study confirm that a significant abatement potential in the agricultural sector can be obtained from different mitigation measures based on the biophysical process underlying crop, livestock, and forest systems, which agrees with the findings of previous studies [20,21,49,51]. Different types of measure such as nitrogen management, carbon sequestration, and livestock feed management make a significant contribution to combat climate change by increasing biomass supply and reducing GHG emissions. Some measures involve synergies between economic and environmental effects, where abatement of GHG emissions does not reduce the profitability of agricultural activities or increase the private benefits of farmers. The results show that the most efficient mitigation measures are optimization of nitrogen fertilization, manure substitution of synthetic fertilizers, crop rotation, and reduction of protein content in the diet of swine. These measures achieve substantial abatement at negative costs (i.e., win-win measures). The cost savings (or benefits) provided by win-win measures may encourage farmers’ endorsement and dispel their concerns. Win-win measures are the focus of ongoing research and policy in some countries [52].

The inclusion of transaction costs and the interactions between measures in the assessment of measures improves the estimation of implementation costs, and the ranking of measures for decision making. In addition, the projection of the agricultural sector developments in Aragon into the future indicates that the volume of emissions could reach 5.2 MtCO_2_e in 2050 if no mitigation efforts are undertaken. The analysis of future scenarios is useful to understand what would occur in coming decades by taking different combination of measures, supporting decision makers in the identification of preferred outcomes.

The implementation of measures depends on the objectives of decision-makers, but also on the availability of biophysical and economic information. The design of measures must take into account local characteristics driving the economic and environmental effects, and social acceptability. Policies have to be legitimate because successful implementation cannot be achieved without the support of stakeholders. The implementation of mitigation measures would require an effective deployment and uptake by farmers and their active cooperation in order to reduce GHG emissions. Cooperation between farmers, stakeholders, and interest groups is needed for a reasonable allocation of farmland resources and for achieving significant nonpoint pollution abatement efforts. Once the mitigation policies are launched, they have to be renegotiated periodically in order to assess their performance and efficiency. Subsequent improvements should incorporate new research results.

The current study has limitations because that MACC representation and cost-efficiencies have been estimated at one point in time. These estimates may become more uncertain in the future because the implementation costs of current measures may change, and because of the development of new measures embodying more advanced technologies. Further advances could be gained by elaborating a more detailed analysis on the viability of mitigation measures. This effort involves setting up mechanisms to ensure the adoption and enforcement of mitigation measures by farmers, taking into account the strategic behavior of interest groups. Understanding the strategic behavior of farmers and other stakeholders is important to advance the required cooperation for successful mitigation.

## 6. Conclusions

This study analyzes a series of mitigation measures in agriculture, giving a first estimation of the potential of individual and combined mitigation measures for agriculture in the region of Aragon (Spain). These mitigation measures rely on information from the biophysical processes underlying crop, livestock, and forest systems, in order to reduce GHG emissions and combat climate change. Comprehensive nutrient management requires knowledge of the sources and sinks of the nitrogen cycle for a correct assessment of measures reducing GHG emissions to the atmosphere, and pollution to water media from crops, manure leaching and runoff. The measures are assessed using the MACC approach, which is an instrument supporting the policy analysis of mitigation measures.

The results of this study reveal the trade-offs and synergies between the economic and environmental effects of mitigation measures, which are used to find out the most efficient mitigation policies in agriculture. The MACC approach includes transaction costs and the interactions between measures, contributing to better decision making on the choice of the appropriate mix of measures. Mitigation scenarios in coming decades also provide policymakers with information on the future outcomes from selected combinations of measures. The results of this study could be of interest for other international locations, especially in semi-arid areas with similar agricultural and climate conditions.

## Figures and Tables

**Table 1:** Description of selected measures.

Measures	Description
**Crop measures**	
**N optimization**	Efficient application of nitrogen fertilization according to the optimal requirements of each crop.
**Crop rotation with legumes**	Crop rotation increases soil carbon sequestration by 0.125 Mg/ha/year and reduces mineral fertilization by 50% [19,53].
**Cover crops**	Cover crops increase carbon sequestration in the soil by 0.35 Mg C/ha/year in woody crops, and reduce greenhouse gas (GHG) emissions by 10% in arable crops [54,55].
**Minimum tillage**	Minimum tillage practices reduce GHG emissions by 0.47 tCO_2_e/ha/year in cereals and 28% in corn [56].
**Nitrification inhibitors**	Adding inhibitors in the soil increases the efficiency of nitrogen use and reduces N_2_O emissions by 30% [54].
**Irrigation modernization**	Replacing surface irrigation by sprinkler and drip systems increases the efficiency of water and nitrogen application.
**Livestock measures**	
**Manure fertilization**	Increasing the share of manure nitrogen fertilization from current 27% to 60%.
**Fat additives**	Adding 1% of fat reduces CH_4_ emissions from enteric fermentation by 4% [44].
**Manure treatment plants**	Manure treatment plants reduce N_2_O emissions by 60% using nitrification and denitrification processes in plants with 50,000 m^3^/year capacity [57]).
**Decrease of protein in the diet of swine**	Adjusting the protein content in feeds reduces nitrogen excretion by 8.5%.
**Forest measures**	
**Forest management**	Adoption of forest management techniques for carbon sequestration.

**Table 2:** Land, animals and nitrogen data used in the calculation of GHG emissions.

Crops	Land (1000 ha)			N Fertilization (kg/ha)			N Leached (kg/ha)		
Dryland		679							
Cereals		558							
Barley		377			77		8		
Wheat		181			45		5		
Fruit trees		121							
Olive		36			60		6		
Vineyard		27			45		5		
Almond		58			45		5		
	Surface	Sprinkler	Drip	Surface	Sprinkler	Drip	Surface	Sprinkler	Drip
Irrigated land	207	125	25						
Cereals									
Barley	74	23		200	150		38	25	
Corn	22	57		450	350		200	120	
Alfalfa	37	33		85	56		32	21	
Wheat	33	12		200	180		48	22	
Rice	7			200			50		
Vegetables									
Pea	4		3	90		60	16		10
Onion	1		1	200		140	33		23
Fruit trees									
Peach	6		7	200		150	60		45
Olive	9		2	120		100	36		30
Vineyard	5		5	120		100	36		30
Almond	6		2	100		80	30		24
Cherry	2		2	100		80	30		24
Apple	1		2	150		130	45		39
Animals	Livestock (1000 heads)			N excreted (kg/animal/year)					
Swine		6309		9					
Sheep		1800		9					
Beef cattle		306		45					
Dairy cattle		15		80					
Chicken		27,283		0.4					

Nitrogen fertilization and the fraction leached by crop have been determined from different sources: [71,72] and information provided by the Soils and Irrigation Department of the Centro de Investigación y Tecnología Agroalimentaria.

## Data Availability

Not applicable.

## Supplementary Materials

The following are available online at https://www.mdpi.com/1660-4601/18/3/1084/s1, Figure S1: Contribution of crops and livestock to agricultural emissions in Aragon, Figure S2: Aragon distribution of GHG emissions from crops and livestock under each measure and forest carbon sequestration, Table S1: Abatement potential and costs in the second scenario, Table S2: Abatement potential and costs in the third scenario.

## References

[1] IPCC (2014). Summary for Policymakers. Climate Change 2014: Impacts, Adaptation, and Vulnerability. Part A: Global and Sectoral Aspects. Contribution of Working Group II to the Fifth Assessment Report of the Intergovernmental Panel on Climate Change.

[2] NASA-GISS (2018). Global Mean Estimates Based on Land and Ocean Data.

[3] Mohammed S., Alsafadi K., Takács I., Harsányi E. (2019). Contemporary changes of greenhouse gases emission from the agricultural sector in the EU-27. Geol. Ecol. Landsc..

[4] IPCC (2007). Climate Change 2007: Synthesis Report. Contribution of Working Groups I, II and III to the Fourth Assessment Report of the IPCC.

[5] IPCC (2014). Climate Change 2014: Synthesis Report. Contribution of Working Group I, II and III to the Fifth Assessment Report of the Intergovernmental Panel on Climate Change.

[6] Liu S., Xie Z., Liu B., Wang Y., Gao J., Zeng Y., Xie J., Xie Z., Jia B., Qin P. (2020). Global river water warming due to climate change and anthropogenic heat emission. Glob. Planet. Chang..

[7] Moragoda N., Cohen S. (2020). Climate-induced trends in global riverine water discharge and suspended sediment dynamics in the 21st century. Glob. Planet. Chang..

[8] Soutter A.R.B., Mõttus R. (2020). Global warming versus climate change: A replication on the association between political self-identification, question wording, and environmental beliefs. J. Environ. Psychol..

[9] Masson-Delmotte V., Zhai P., Pörtner H.O., Roberts D., Skea J., Shukla P.R., IPCC (2018). Global Warming of 1.5 °C. An IPCC Special Report on the Impacts of Global Warming of 1.5 °C above Pre-industrial Levels and Related Global Greenhouse Gas Emission Pathways, in the Context of Strengthening the Global Response to the Threat of Climate Change, Sustainable Development, and Efforts to Eradicate Poverty.

[10] UNEP (2019). Global Environment Outlook Geo-6, Healthy Planet, Healthy People.

[11] UNFCCC (2015). Adoption of the Paris Agreement.

[12] MAPAMA (2017). Inventory of Greenhouse Gas Emissions in Spain, Series 1990–2015. Summary Report. Secretary of State for the Environment, General Directorate for Environmental and Natural Quality and Assessment, General Sub-Directorate for Air Quality and Industrial Environment.

[13] Smith P. (2012). Soils and climate change. Curr. Opin. Environ. Sustain..

[14] Hammad H.M., Nauman H.M.F., Abbas F., Ahmad A., Bakhat H.F., Saeed S., Shah G.M., Ahmad A., Cerdà A. (2020). Carbon sequestration potential and soil characteristics of various land use systems in arid region. J. Environ. Manag..

[15] Ingram J., Mills J., Frelih-Larsen A., Davis M., Merante P., Ringrose S., Molnar A., Sánchez B., Ghaley B.B., Karaczun Z. (2014). Managing Soil Organic Carbon: A Farm Perspective. EuroChoices.

[16] Kahil T., Albiac J. (2012). Instrumentos de política de cambio climático en la agricultura de Aragón. Rev. Española Estud. Agrosoc. Pesq..

[17] Kahil T., Albiac J. (2013). Greenhouse gases mitigation policies in the agriculture of Aragon, Spain. Bio-Based Appl. Econ..

[18] Plaza-Bonilla D., Alvaro-Fuentes J., Arrúe J.L., Cantero-Martínez C. (2012). Tillage and nitrogen fertilization effects on nitrous oxide yield-scaled emissions in a rainfed Mediterranean area. Agric. Ecosyst. Environ..

[19] Sánchez B., Iglesias A., McVittie A., Álvaro-Fuentes J., Ingram J., Mills J., Lesschen J.P., Kuikman P.J. (2016). Management of agricultural soils for greenhouse gas mitigation: Learning from a case study in NE Spain. J. Environ. Manag..

[20] Albiac J., Kahil T., Notivol E., Calvo E. (2017). Agriculture and climate change: Potential for mitigation in Spain. Sci. Total Environ..

[21] Pellerin S., Bamière L., Angers D., Béline F., Benoit M., Butault J.P., Chenu C., Colnenne-David C., De Cara S., Delame N. (2017). Identifying cost-competitive greenhouse gas mitigation potential of French agriculture. Environ. Sci. Policy.

[22] CEDEX (Centro de Estudios y Experimentación de Obras Públicas) (2017). Evaluación del Impacto del Cambio Climático en los Recursos Hídricos y Sequías.

[23] Gobierno de Aragón (2019). Calidad del agua de consumo humano en la Comunidad Autónoma de Aragón.

[24] ESPON (2012). Climate Change and Europe’s Regions. Featured Map. ESPON Climate Project Co-Financed by the European Regional Development Funds.

[25] IAEST (2018). Superficie de Aragón.

[26] MAPAMA (2017). Encuesta Sobre Superficies y Rendimientos de Cultivos: Informe Sobre Regadíos en España (ESYRCE).

[27] Gobierno de Aragon (2017). La Producción Agraria en Aragón, Documento de Síntesis.

[28] Smith S., Braathen N. (2015). Monetary Carbon Values in Policy Appraisal: An Overview of Current Practice and Key Issues. Environ. Work. Pap..

[29] IPCC (2006). Agricultura, Silvicultura y Otros Usos de la Tierra, Capítulo 1: Introducción. Directrices del IPCC de 2006 Para los Inventarios Nacionales de Gases de Efecto Invernadero.

[30] IPCC (2019). 2019 Refinement to the 2006 IPCC Guidelines for National Greenhouse Gas Inventories.

[31] EEA (2014). CH4 Emission Factors.

[32] IPCC (2006). Agricultura, Capitulo 4: Orientación del IPCC sobre las Buenas Prácticas y la Gestión de la Incertidumbre en los Inventarios Nacionales de Gases de Efecto Invernadero.

[33] Moran D., MacLeod M., Wall E., Eory V., Pajot G., Matthews R., McVittie A., Barnes A., Rees B., Moxey A. (2008). UK Marginal Abatement Cost Curves for the Agriculture and Land Use, Land-Use Change and Forestry Sectors out to 2022, with Qualitative Analysis of Options to 2050.

[34] Schulte R., Crosson P., Donnellan T., Farrelly N., Finnan J., Lanigan G., O’Brien D., Shalloo L., Thorne F. (2012). A Marginal Abatement Cost Curve for Irish Agriculture.

[35] Crespo D., Albiac J., Kahil T., Esteban E., Baccour S. (2019). Tradeoffs between Water Uses and Environmental Flows: A Hydroeconomic Analysis in the Ebro Basin. Water Resour. Manag..

[36] Baccour S., Albiac J., Esteban E. Modelización Hidroeconómica de la Contaminación Difusa y la Escasez de Agua en la Cuenca del Ebro. https://citarea.cita-aragon.es/citarea/bitstream/10532/5064/3/2020_213.pdf.

[37] Asgedom H., Kebreab E. (2011). Beneficial management practices and mitigation of greenhouse gas emissions in the agriculture of the Canadian Prairie: A review. Agron. Sustain. Dev..

[38] Burney J.A., Davis S.J., Lobell D.B. (2010). Greenhouse gas mitigation by agricultural intensification. Proc. Natl. Acad. Sci. USA.

[39] Wilhelm W.W., Johnson J.M.F., Hatfield J.L., Voorhees W.B., Linden D.R. (2004). Crop and soil productivity response to corn residue removal. J. Agron..

[40] Kell D.B. (2012). Large-scale sequestration of atmospheric carbon via plant roots in natural and agricultural ecosystems: Why and how. Philos. Trans. R. Soc. B.

[41] Poeplau C., Don A. (2015). Carbon sequestration in agricultural soils via cultivation of cover crops: A meta-analysis. Agric. Ecosyst. Environ..

[42] Ogle S.M., Breidt F.J., Paustian K. (2005). Agricultural management impacts on soil organic carbon storage under moist and dry climatic conditions of temperate and tropical regions. Biogeochemistry.

[43] Thornton P.K., Herrero M. (2010). Potential for reduced methane and carbon dioxide emissions from livestock and pasture management in the tropics. Proc. Natl. Acad. Sci. USA.

[44] Martin C., Morgavi D., Doreau M. (2010). Methane mitigation in ruminants: From microbe to the farm scale. Animal.

[45] Herrero M., Henderson B., Havlík P., Thornton P.K., Conant R.T., Smith P., Wirsenius S., Hristov A.N., Gerber P., Gill M. (2016). Greenhouse gas mitigation potentials in the livestock sector. Nat. Clim. Chang..

[46] Bravo F. (2007). El Papel de los Bosques Españoles en la Mitigación del Cambio Climático.

[47] Ruiz-Peinado R., Bravo-Oviedo A., López-Senespleda E., Bravo F., Río M. (2017). Forest management and carbon sequestration in the Mediterranean region: A review. For. Syst..

[48] McCarl B.A., Schneider U.A. (2000). U.S. Agriculture’s Role in a Greenhouse Gas Emission Mitigation World: An Economic Perspective. Appl. Econ. Perspect Policy.

[49] MacLeod M., Moran D., Eory V., Rees R.M., Barnes A., Topp C.F.E., Ball B., Hoad S., Wall E., McVittie A. (2010). Developing greenhouse gas marginal abatement cost curves for agricultural emissions from crops and soils in the UK. Agric. Syst..

[50] Moran D., Macleod M., Wall E., Eory V., McVittie A., Barnes A., Rees R., Topp C.F.E., Moxey A. (2011). Marginal Abatement Cost Curves for UK Agricultural Greenhouse Gas Emissions. J. Agric. Econ..

[51] Wang W., Koslowski F., Nayak D.R., Smith P., Saetnan E., Ju X., Guo L., Han G., Perthuis C.D., Lin E. (2014). Greenhouse gas mitigation in Chinese agriculture: Distinguishing technical and economic potentials. Glob. Environ. Chang..

[52] Moran D., Lucas A., Barnes A. (2013). Mitigation win–win. Nat. Clim. Chang..

[53] Lal R., Bruce J.P. (1999). The potential of world cropland soils to sequester C and mitigate the greenhouse effect. Environ. Sci. Policy.

[54] Sanz-Cobena A., Lassaletta L., Aguilera E., Prado A., Garnier J., Billen G., Iglesias A., Sánchez B., Guardia G., Abalos D. (2017). Strategies for greenhouse gas emissions mitigation in Mediterranean agriculture: A review. Agric. Ecosyst. Environ..

[55] González-Sánchez E.J., Ordóñez-Fernández R., Carbonell-Bojollo R., Veroz-González O., Gil-Ribes J.A. (2012). Meta-analysis on atmospheric carbon capture in Spain through the use of conservation agriculture. Soil Tillage Res..

[56] Forte A., Fiorentino N., Fagnano M., Fierro A. (2017). Mitigation impact of minimum tillage on CO_2_ and N_2_O emissions from a Mediterranean maize cropped soil under low-water input management. Soil Tillage Res..

[57] Teresa M., Herrero E., Bescós B. (2016). Evaluación de Sistemas de Gestión de Estiércol en Europa. Resultados del Proyecto LIFE-MANEV.

[58] Liu Z., Shen J. (2006). Measuring Transaction Costs: Theoretic Development and Application. Financ. Trade Econ..

[59] Cheung S. (1998). The Transaction Costs Paradigm: 1998 Presidential Address, Western Economic Association. Econ. Inq..

[60] Dahlman C.J. (1979). The Problem of Externality. J. Law Econ..

[61] McCann L. (2013). Transaction costs and environmental policy design. Ecol. Econ..

[62] Garrick D. (2013). Transaction costs and environmental policy: Taking stock, looking forward. Ecol. Econ..

[63] Howitt R.E. (1994). Empirical analysis of water market institutions: The 1991 California water market. Resour. Energy Econ..

[64] McCann L.M.J., Easter K.W. (2000). Estimates of public transaction costs in NRCS programs. J. Agric. Appl. Econ..

[65] Mettepenningen E., Verspecht A., Huylenbroeck G.V. (2009). Measuring private transaction costs of European agri-environmental schemes. J. Environ. Plan. Manag..

[66] Coggan A., Whitten S.M., Bennett J. (2010). Influences of transaction costs in environmental policy. Ecol. Econ..

[67] Rorsted P., Vatn A., Kvakkestad V. (2007). Why do transaction costs of agricultural policies vary?. Agric. Econ..

[68] Kahil T. (2011). Instrumentos de Mitigación y Adaptación al Cambio Climático en la Agricultura de Aragón. Master’s Thesis.

[69] Cui Z., Zhang H., Chen X., Zhang C., Ma W., Huang C., Zhang W., Mi G., Miao Y., Li X. (2018). Pursuing sustainable productivity with millions of smallholder farmers. Nature.

[70] Clark M.A., Domingo N.G.G., Colgan K., Thakrar S.K., Tilman D., Lynch J., Azevedo I.L., Hill J.D. (2020). Global food system emissions could preclude achieving the 1.5° and 2°C climate change targets. Science.

[71] Orús F. (2006). Fertilización Nitrogenada: Guía de Actualización. Informaciones Técnicas, Número Extraordinario.

[72] Mema M. (2006). Las Políticas de Control de la Contaminación Difusa en el Valle Medio del Ebro. Ph.D. Thesis.

[73] Conniff R. (2019). Scrubbing Carbon from the Sky: Can we remove enough CO_2_ from the atmosphere to slow or even reverse climate change?. Sci. Am..

[74] Albiac J., Soriano J.S., Dinar A., Albiac J., Soriano J.S., Dinar A. (2008). Game theory: A useful approach for policy evaluation in natural resources and the environment. Game Theory and Policymaking in Natural Resources and the Environment.

[75] Jiao X., Lyu Y., Wu X., Li H., Cheng L., Zhang C., Yuan L., Jiang R., Jiang B., Rengel Z. (2016). Grain production versus resource and environmental costs: Towards increasing sustainability of nutrient use in China. J. Exp. Bot..

