# Supplementary material for: Cost-Effective Mitigation of Greenhouse Gas Emissions in the Agriculture of Aragon, Spain
Source: Int J Environ Res Public Health. 2021 Jan 26;18(3):1084. doi: 10.3390/ijerph18031084 (PMC7908559; doi:10.3390/ijerph18031084)
Supplement: Supplementary file 1 [file ijerph-18-01084-s001.pdf]

Cost-effective mitigation of greenhouse gas emissions in the agriculture of Aragon, Spain

Safa Baccour [1], Jose Albiac [2,] *, Taher Kahil [2]

1 Department of Agricultural Economics, CITA-IA2, Saragossa, Spain; baccour.safa@gmail.com
2 International Institute for Applied Systems Analysis (IIASA), Laxenburg, Austria; kahil@iiasa.ac.at (Taher Kahil), maella@unizar.es (Jose Albiac).
* Correspondence: maella@unizar.es

Supplementary materials

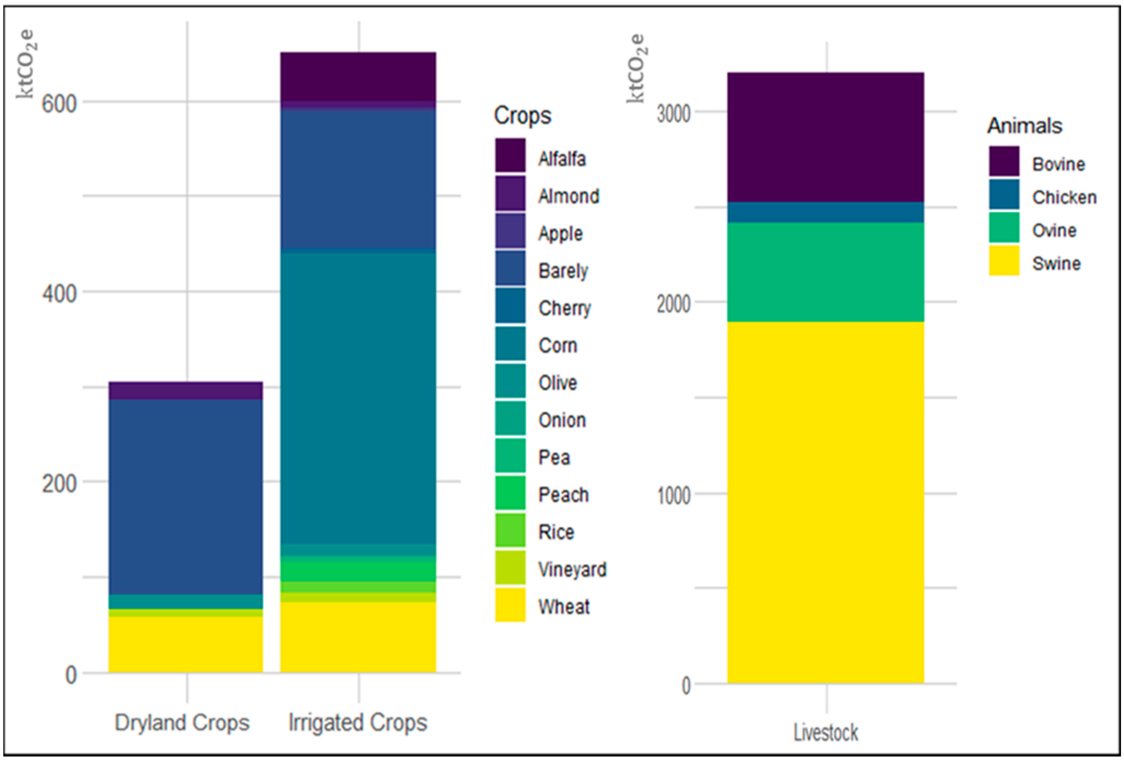

Figure S1. Contribution of crops and livestock to agricultural emissions in Aragon.

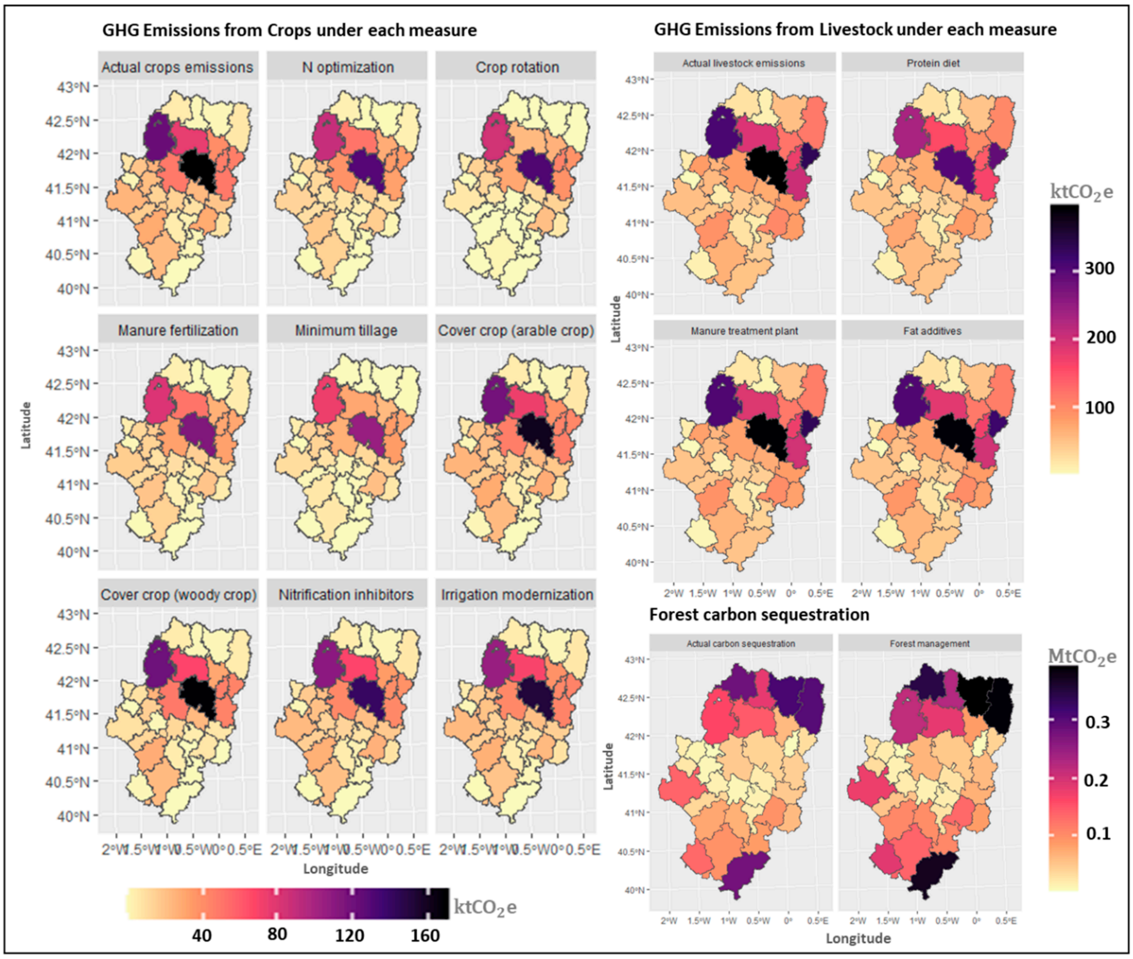

Figure S2. Aragon distribution of GHG emissions from crops and livestock under each measure and forest carbon sequestration.

Table S1. Abatement potential and costs in the second scenario.

	Individual measures			Combined measures		
GHG emissions in 2050	**5,2**			**5,2**		
	Measures implementation					
Measures	**AP[1] ($MtCO_2e$)**	**Private cost (M€)**	**Private cost with TC[2] (M€)**	**AP ($MtCO_2e$)**	**Private cost (M€)**	**Private cost with TC (M€)**
**N optimization**	0,29	-38	-30	0,29	-38	-30
**Manure fertilization**	0,32	-11	-5	0,22	-7	-4
**Minimum tillage**	0,41	-11	1	0,39	-11	1
GHG emissions and costs with measures implementation	**4,2**	**-60**	**-34**	**4,3**	**-56**	**-33**

[1]: AP, Abatement potential. [2]: TC, Transaction costs

Table S2. Abatement potential and costs in the third scenario.

	Individual measures			Combined measures		
GHG emissions in 2050		**5,2**		**5,2**		
	Measures implementation					
Measures	**AP ($MtCO_2e$)**	**Private cost (M€)**	**Private cost with TC (M€)**	**AP ($MtCO_2e$)**	**Private cost (M€)**	**Private cost with TC (M€)**
**N optimization**	0,29	-38	-30	0,29	-38	-30

**Manure fertilization**	0,32	-11	-5	0,22	-7	-4
**Crop rotation**	0,39	-21	-16	0,32	-10	7
**Minimum tillage**	0,41	-11	1	0,39	-11	1
**Protein diet**	0,54	-7	-5	0,54	-7	-5
**Forest management**	0,9	-3	0,5	0,9	-3	0,5
**Cover crop (woody crop)**	0,03	0,2	1	0,16	7	8
**Cover crop (arable crop)**	0,16	7	8	0,02	2	2
**Manure treatment plant**	0,08	11	14	0,07	9	12
**Nitrification inhibitor**	0,11	8	10	0,04	8	10
**Irrigation modernization**	0,1	18	34	0,05	23	39
**Fat additives (Bovine)**	0,05	28	34	0,05	28	34
**Fat additives (Ovine)**	0,06	158	182	0,06	158	182
GHG emissions and costs with measures implementation	**1,8**	**139**	**228**	**2,1**	**159**	**257**
